# A Stacked BiLSTM Neural Network Based on Coattention Mechanism for Question Answering

**DOI:** 10.1155/2019/9543490

**Published:** 2019-08-21

**Authors:** Linqin Cai, Sitong Zhou, Xun Yan, Rongdi Yuan

**Affiliations:** Key Laboratory of Industrial Internet of Things and Networked Control, Ministry of Education, Chongqing University of Posts and Telecommunications, Chongqing 400065, China

## Abstract

Deep learning is the crucial technology in intelligent question answering research tasks. Nowadays, extensive studies on question answering have been conducted by adopting the methods of deep learning. The challenge is that it not only requires an effective semantic understanding model to generate a textual representation but also needs the consideration of semantic interaction between questions and answers simultaneously. In this paper, we propose a stacked Bidirectional Long Short-Term Memory (BiLSTM) neural network based on the coattention mechanism to extract the interaction between questions and answers, combining cosine similarity and Euclidean distance to score the question and answer sentences. Experiments are tested and evaluated on publicly available Text REtrieval Conference (TREC) 8-13 dataset and Wiki-QA dataset. Experimental results confirm that the proposed model is efficient and particularly it achieves a higher mean average precision (MAR) of 0.7613 and mean reciprocal rank (MRR) of 0.8401 on the TREC dataset.

## 1. Introduction

Deep learning forms a more abstract high-level representation attribute feature by combining low-level features to discover the distributed feature representations of data. It provides an effective method for NLP research. In recent years, intelligent question answering in the NLP field has emerged as a prominent discipline research hotspot in both academia and industry, which has been widely used by many influential question answering systems. Answer selection plays a vital role in question answering task, and it mainly encodes QA pair and inputs them into the model to extract the key information and get the corresponding representation [1]. Thus, the main task is to map the question and answer sentences into a joint feature space to generate the codependent representation for them. In the end, an algorithm is utilized to calculate their similarity.

In the past few years, most question answering studies [2–4] were based on knowledge bases and FAQs, which use machine learning to analyze and retrieve keywords. Unfortunately, both of them lack relevant semantic analysis of the questions and answers, which results in a shortcoming of strong artificial dependency and poor scalability.

With the significant innovation of deep learning, deep neural networks are able to availably map the meaning of a single word in a sentence to a continuous representation of the entire sentence, and the meaning of the sentence representation obtained is more complete. Because deep learning reduces the need for manual feature engineering and adapting to new tasks, it has become an important research method for various tasks of NLP in the last several years, and a large number of researchers take advantage of its end-to-end model for sentence semantic analysis to implement question answering tasks. Feng et al. [5] and Wang and Nyberg [6] resorted convolutional neural networks (CNN) and Bidirectional Long Short-Term Memory Networks to capture single sentence semantics, respectively. Nevertheless, both of them ignored the interrelationship between encoded representations of question and answer. Recently, the model based on the attention mechanism has been explored for question answering. Tan et al. [7] and Nie et al. [8] proposed a BiLSTM model that combines the attention mechanism to construct a better answer representation according to the input question sentences. The model takes the effect of the question on the answer list encoding into account, but they ignore the effect of the answer on the encoding representation of the question, which will cause some deviations in the final prediction result. For instance, the question 1 is “Michael, what are you eating?” and the question 2 is “Michael, why are you eating so much?” and the answer is “Yeah, I'm eating a hamburger.” The words “what” & “eating” in question 1 and the words “I'm” & “eating hamburger” in answer have a certain semantic association, and we could easily infer that the answer is corresponding to the question 1. It means that each answer has some intrinsic connection with the question, and to some extent, the question representation is affected by different answers. In addition to analyzing the answers from the questions, we can also infer some results about the questions from the answers.

In this paper, we construct a deep learning architecture for question answering, where questions and answers are limited to a single sentence. The cores of our architecture are two distributed sentence models working in parallel, based on a stacked BiLSTM neural network. We map questions and answers to the corresponding distribution vectors and finally calculate the semantic similarity between them. BiLSTM neural networks have been widely used in recent years to deal with NLP issues [9–11]. Zhang and Ma [12] established a new deep learning model based on BiLSTM networks to accomplish the answer selection task and achieved favorable results. Motivated by this work, we utilize the stacked BiLSTM deep neural network that incorporates the coattention mechanism to semantically understand and model the QA pair, thus allowing model to capture long dependency sentence-level features and generate deeper codependent representations for the QA pair. Additionally, the cosine similarity and the Euclidean distance are reconciled as a new metric to measure the semantic similarity and distance between the questions and the answers. Experiments are settled on the Text REtrieval Conference 8-13 QA dataset and Wiki-QA dataset. Comparison shows that our experimental model achieved the best experimental results.

The main contributions of this paper are summarized as follows:

- A stacked BiLSTM neural network is resorted to attain the vector representation of the input sentence, which can effectively capture the semantics of the sentence.
- Our model combines coattention mechanism and attention mechanism to encode sentences to obtain the interaction and influence between the QA pair.
- The cosine similarity and the Euclidean distance are reconciled to calculate the degree of matching between two vectors. This method is able to take the distance and angle relationship between vectors into consideration.

The rest of this paper is organized as follows.  Section 2 gives a brief review of related work.  Section 3 presents the proposed framework and method for question answering.  Section 4 is a detailed analysis and summary of the experimental results. We will draw a conclusion and discuss the next work in Section 5.

## 2. Related Work

Research in question answering has been greatly boosted by the Text REtrieval Conference series since 1999. Recently, a number of related works [12–15] have proposed many efficient models for question answering. We compare and correlate the proposed stacked BiLSTM neural networks, coattention mechanism, and scoring metric with our other methods in the literature as follows.

### 2.1. Long Short-Term Memory Neural Networks

Previously, traditional research approaches concentrated on syntactic matching between the questions and answers. Punyakanok et al. [3] was the earliest to propose the general question and answer matching model via dependency tree models. Later, both Heilman and Smith [2] and Khan et al. [16] presented a probabilistic tree edit algorithm to model sentence. Yao et al. [17] constructed a linear-chain conditional random field based on TREC-QA dataset, which extracted the answer as the answer sequence labeling problem of the tree editing sentence. Moreover, Zhou et al. [4] resorted lexical model based on word relations to select answer sentences. But these traditional models rely excessively on external conditions such as manual labeling of information, which requires a large amount of related work to achieve.

In the recent work of question answering, the mainstream is based on deep learning methods. Yih et al. [18] and Wang et al. [19] developed a semantic parsing framework by a semantic similarity model using convolutional neural networks. Wang and Nyberg [6] used a stacked BiLSTM network to sequentially read words from the question and answer sentences, which did not require any syntactic parsing or external knowledge resources such as WordNet. However, these models failed to consider the codependent representations of the questions and answers. Thus, we add attention mechanism to the deep neural networks to capture the associations between the QA pair.

### 2.2. Coattention Mechanism

The attention mechanism is appropriate for inferring the mapping relationship between different modal data extremely. It can help a framework like a codec to properly acquire the interrelationships of multiple content models, thus expressing more effectively [1]. There are plenty of related works having explored the attention mechanism in question answering. Based on bidirectional recurrent neural networks, Bahdanau et al. [20] added the attention mechanism to the model to encode and decode the sentence in machine translation. Zhang et al. [21] examined inner attention mechanism and outer attention mechanism in discourse representation for implicit discourse relation recognition. The result showed a marvelous improvement on marco-*F*1 point is 1.61%. Inspired by the related work in Bahdanau et al. [20] and Fu et al. [22], Tan et al. [7] and Xiang et al. [23] successively proposed an attention mechanism based on bidirectional single-layer LSTMs for question-answer matching, which is able to construct better answer representations according to the input question. Meanwhile, Lu et al. [24] took the lead in presenting a hierarchical coattention model for visual question answering. They used the coattention mechanism to compute a conditional representation of the image given the question and a conditional representation of the question given the image. Enlightened by this work, Xiong et al. [10] presented a dynamic coattention network (DCN) to obtain the codependent representations of question and document, and they used a dynamic point decoder to sort potential answers. The experiment achieved 0.8% EM and 2.1% *F*1 improvement on SQuAD dataset. A more refined coattention model was proposed by Zhang and Ma [12]. The author combined the coattention mechanism with the attention mechanism to encode the representation of questions and answers, and this model significantly utilized the inner relationship between questions and answers to enhance the experiment results. Our research also adopts a similar coattention mechanism to extract the statement features.

### 2.3. Scoring Mechanism

In many previous works such as Liu [25] and He et al. [26], cosine similarity has been proven to be an effective metric for evaluating the similarity between two chord vectors, and it has been widely used in complex queries and matching in recent years. However, Lee et al. [27] resorted the Euclidean distance as the classification decision-making function to measure the average distance between the new data point and the support vectors from different categories, and the data showed that it is efficient. Feng et al. [5] proposed two novel metrics GESD (Geometric mean of Euclidean and Sigmoid Dot product) and AESD (Arithmetic mean of Euclidean and Sigmoid Dot product) in their answer selection task. They proposed two metrics that are the best among all the comparison metrics. In the work of Yin et al. [15], the cosine similarity and the Euclidean distance were separately used to calculate the sentence similarity and measure the semantic distance between different sentences. The result revealed that the simultaneous use of two evaluation mechanisms is superior to using only cosine similarity metric. Unlike the previous research, our approach improves and optimizes previous methods by reconciling the two functions. Our results show that the method is efficient.

## 3. Proposed Question Answering Model

**Figure 1:**
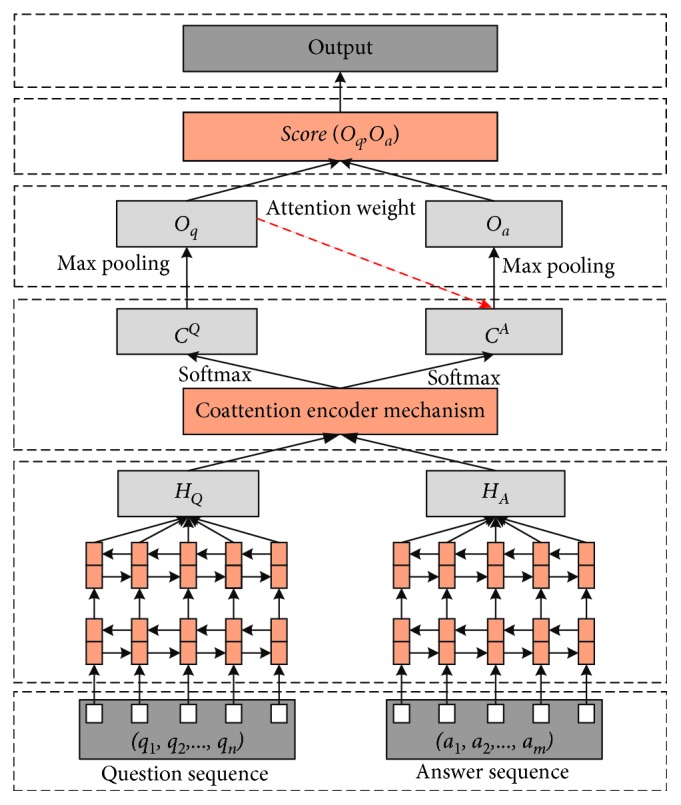
Framework of the proposed neural network model.

In this section, we describe the proposed question answering model based on deep learning, which is optimized based on the architecture of Tan et al. [1] and Xiong et al. [10]. The overview of the framework is constructed in Figure 1.

In Figure 1, we first utilize the pretrained GloVe to construct word embedding layer, and this word embedding provides the vector representation for each question and its candidate answers. Second, the stacked BiLSTM neural network serves as an encoder that extracts hidden features from each input sentence. Corresponding representations can be obtained by the questions based on the coattention mechanism. After entering the question vector into the maximum pooling, the attention mechanism is used to generate an answer embedding according to the question representation. At last, we combine cosine similarity and Euclidean distance to measure the degree of matching between the question vector and the answer vector.

### 3.1. A Stacked BiLSTM Neural Network

**Figure 2:**
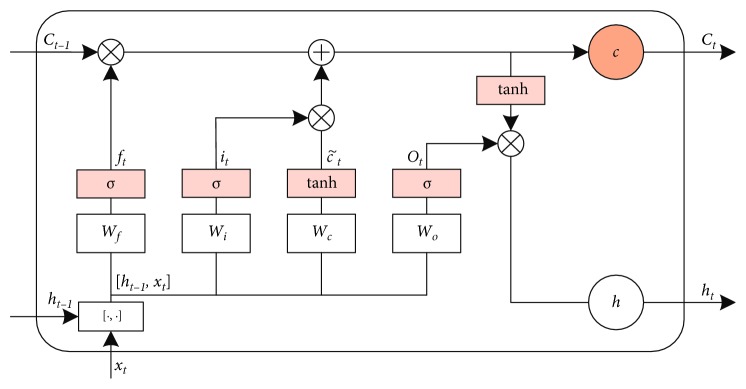
Architecture of Long Short-Term Memory cell.

LSTM networks architecture was originally developed by Hochreiter and Schmidhuber [28]. More formally, an input sequence vector *x*=(*x*_1_, *x*_2_,…, *x*_*n*_) is given, where *n* indicates the length of the input sentence. The core structure of the LSTM is the use of three control gates to control a memory cell activation vector *c*. The first forget gate determines how much of the cell state *c*_*t*−1_ at the previous time is retained until the current cell state *c*_*t*_; the second input gate determines the extent to which the input *x*_*t*_ of the network is saved to the current cell state *c*_*t*_; the third output gate determines how much of the cell state *c*_*t*_ is transmitted to the current output value *h*_*t*_ of the LSTM networks. The three gates are a fully connected layer, and its input is a vector and the output is a real number in [0, 1]. The basic LSTM cell architecture is shown in Figure 2, and its representation is as follows:

(1) $$\begin{array}{c} \text{Input gates}:i_{t}=\sigma \left(W_{ix}x_{t}+W_{ih}h_{t-1}+b_{i}\right),\\ \text{Forget gates}:f_{t}=\sigma \;\left(W_{fx}x_{t}+W_{fh}h_{t-1}+b_{f}\right),\\ \text{Output gates}:o_{t}=\sigma \left(W_{ox}x_{t}+W_{oh}h_{t-1}+b_{o}\right),\\ \text{Cell states}:c_{t}=f_{t}\;*\;c_{t-1}+i_{t}\;*\;\tanh\left(W_{cx}x_{t}+W_{ch}h_{t-1}+b_{c}\right),\\ \text{Cell outputs}:h_{t}=o_{t}\;*\;\tanh\left(c_{t}\right), \end{array}$$

where *σ* is the logistic sigmoid function, *x*_*t*_ indicates *t*-th word vector of the sentence and *h*_*t*_ indicates the hidden state, *W* terms and *b* terms, respectively, represent weight matrices (e.g., *W*_*xf*_ represents the forget gate weight matrix) and bias vectors (e.g., *b*_*i*_ represents the input gate bias vector) for the three gates.

To overcome the shortcoming of single LSTM cell that can only capture previous context but not utilize the future context, Schuster and Paliwal [29] invented bidirectional recurrent neural networks (BRNN) to combine two separate hidden LSTM layers of opposite directions to the same output. With this structure, the output layer is able to utilize related information from both the previous and future context. A BiLSTM calculates the input sequence *x*=(*x*_1_, *x*_2_,…, *x*_*n*_) from the opposite direction to a forward hidden sequence ${\vec{h}}_{t}=\left({\vec{h}}_{1},{\vec{h}}_{2},\ldots ,{\vec{h}}_{n}\right)$ and a backward hidden sequence ${\overset{⟵}{h}}_{t}=\left({\overset{⟵}{h}}_{1},{\overset{⟵}{h}}_{2},\ldots ,{\overset{⟵}{h}}_{n}\right)$. The encoded vector *y*_*t*_ is formed by the concatenation of the final forward and backward outputs, $y_{t}=\left[{\vec{h}}_{t},{\overset{⟵}{h}}_{t}\right]$.

(2) $$\begin{array}{c} {\vec{h}}_{t}=\sigma \;\left(W_{\vec{h}x}x_{t}+W_{\vec{h}\vec{h}}{\vec{h}}_{t-1}+b_{\vec{h}}\right),\\ {\overset{⟵}{h}}_{t}=\sigma \;\left(W_{\vec{h}x}x_{t}+W_{\vec{h}\vec{h}}{\overset{⟵}{h}}_{t+1}+b_{\vec{h}}\right),\\ y_{t}=W_{y\vec{h}}{\vec{h}}_{t}+W_{y\vec{h}}{\overset{⟵}{h}}_{t}+b_{y}, \end{array}$$

where *y*=(*y*_1_, *y*_2_,…*y*_*t*_ …, *y*_*n*_) is the output sequence of the first hidden layer.

**Figure 3:**
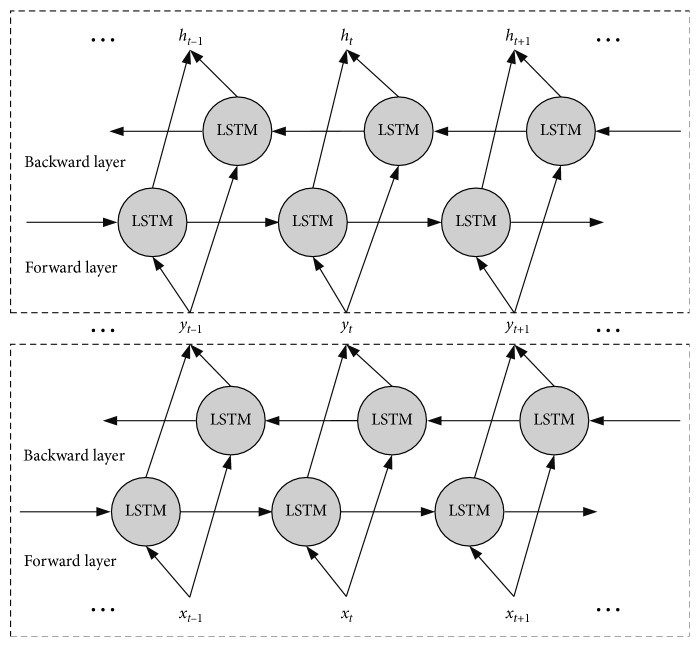
Architecture of the stacked BiLSTM networks.

Some previous works represented that by stacking multiple BiLSTM in neural networks, the performance of classification or regression can be further improved [30–32]. Moreover, there is some related theoretical support to show that a deep hierarchical model is more efficient in representing some functions than a shallow one [6, 33]. We have defined a stacked BiLSTM network where the output *y*_*t*_ from the lower layer becomes the input of the upper layer. The stacked BiLSTM structure is illustrated in Figure 3:

(3) $$\begin{array}{c} h_{t}=W_{h\dot{h}}{\vec{h}}_{t}+W_{h\dot{h}}{\overset{⟵}{h}}_{t}+b_{h}. \end{array}$$

Defining *Q*=(*q*_1_, *q*_2_,…, *q*_*n*_) and *A*=(*a*_1_, *a*_2_,…, *a*_*m*_) to represent question sequences and answer sequences, respectively, where *n* and *m* indicate the length of the questions and answers, and *q*_*t*_ and *a*_*t*_ indicate the *t*-th words of the questions and answers. We run a stacked BiLSTM over the questions and answers to obtain their hidden state matrixes *H*_Q_ and *H*_A_, and the mathematics is as follows:

(4) $$\begin{array}{c} h_{t}^{q}=\text{sBiLSTM}\left(h_{t-1}^{q},h_{t+1}^{q},q_{t}\right),\;h_{0}^{q}=0,\\ h_{t}^{a}=\text{sBiLSTM}\left(h_{t-1}^{a},h_{t+1}^{a},a_{t}\right),\;h_{0}^{a}=h_{n}^{q},\\ H_{\text{Q}}=\left[h_{1}^{q},h_{2}^{q},\ldots ,h_{n}^{q}\right]\in R^{d*n},\\ H_{\text{A}}=\left[h_{1}^{a},h_{2}^{a},\ldots ,h_{m}^{a}\right]\in R^{d*m}, \end{array}$$

where *d* is the dimension of the hidden state.

### 3.2. Coattention Mechanism for Question Representation

**Figure 4:**
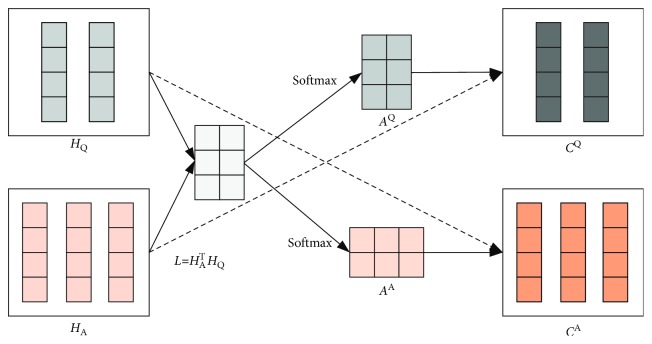
An illustration of the coattention mechanism.

Here, we implement a coattention mechanism to encode question according to the answer sequences, as shown in Figure 4. Motivated by the work of Xiong et al. [10], we try to enforce more question-answer interactions by designing more careful matrix multiplication, operations, and concatenations in the coattention mechanism.

We first perform matrix multiplication to calculate the affinity matrix *L*, which includes affinity scores corresponding to all pairs of question and answer words. It can be described as follows:

(5) $$\begin{array}{c} L=H_{\text{A}}^{\text{T}}H_{\text{Q}}\in R^{m*n}. \end{array}$$

Softmax function is applied to standardize vector elements, and it is effective in dealing with multiclassification and probability distribution problems. Hence, the column- and row-based softmax functions are utilized to generate attention weights for the hidden states of question and answer separately in the following equation:

(6) $$\begin{array}{c} A^{\text{Q}}=\text{soft}\max\left(L\right)\in R^{m*n},\\ A^{\text{A}}=\text{soft}\max\;\left(L^{\text{T}}\right)\in R^{n*m}. \end{array}$$

In order to obtain the attention vector of the question in light of each word of answers, we concatenate attention weights and affinity matrix to compute new context vectors *C*^Q^ and *C*^A^. Here, *C*^Q^ and *C*^A^ are the results of the interaction between the question and the answer vector:

(7) $$\begin{array}{c} C^{\text{Q}}=H_{\text{A}}A^{\text{Q}}\in R^{d*n},\\ C^{\text{A}}=H_{\text{Q}}A^{\text{A}}\in R^{d*m}. \end{array}$$

### 3.3. Attentive Attention Mechanism for Answer Representation

To reduce the information loss of stacked BiLSTM, a soft attention flow layer can be used for linking and integrating information from the question and answer words [1, 13]. In the proposed model, the attention mechanism is applied to the output of coattention. We assume that *C*_*t*_^Q^ indicates *t*-th attention context vector of the question, and the max pooling is taken to convert the input into a fixed-length vector output *O*_q_. Then, the softmax weights of all context vectors (*C*_1_^A^, *C*_2_^A^,…, *C*_*m*_^A^) can be learned autonomously according to *O*_q_ via the attention mechanism, and the weighted context vector *O*_a_ of the answer is used as the final representation:

(8) $$\begin{array}{c} O_{\text{q}}=\underset{0<t<=n}{\max}C_{t}^{\text{Q}},\\ M_{\text{aq}}\left(t\right)=\tanh\;\left(W_{am}C_{t}^{\text{A}}+W_{qm}O_{\text{q}}\right),\\ S_{\text{aq}}\left(t\right)\;\propto \;\exp\;\left(w_{ms}^{\text{T}}M_{\text{aq}}\left(t\right)\right),\\ O_{\text{a}}=\overset{m}{\underset{t=1}{\sum }}C_{t}^{\text{A}}S_{\text{aq}}\left(t\right). \end{array}$$

Here, *W*_*am*_ and *W*_*qm*_ represent the attention matrices of *C*_*t*_^A^ and *O*_q_, respectively. *w*_*ms*_ denotes the attention weight vector. The final representation *O*_a_ of answer is determined by the attention weight *S*_aq_(*t*) for answer context vector of the *t*-th word. It is normalized by the softmax function, which is proportional to *C*_*t*_^A^. Higher values for *S*_aq_(*t*) indicate higher correlation between *C*_*t*_^A^ and the question, and the question vector will get more attention.

### 3.4. Answer Scoring Mechanism and Objective Function

**Figure 5:**
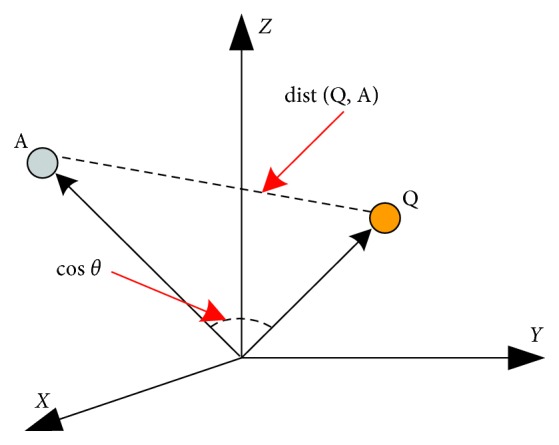
Schematic diagram of cosine similarity and Euclidean distance.

In this work, we resort a method to reconcile cosine similarity and Euclidean distance to evaluate the degree of matching between the questions and answers. Cosine similarity represents the angle between two vectors, and the Euclidean distance represents the distance between two points in Euclidean space. We hope that the distance between the question and the answer semantic vector to be close enough and the angle is small enough, to maximize the similarity calculation between question and answer pair sentence vectors. The schematic diagram of cosine similarity and Euclidean distance is shown in Figure 5.

A vector representation of the question and answer is obtained from the hidden layer of the model. The cosine similarity and Euclidean distance calculation details are as below. Score(*O*_q_, *O*_a_) is the final match result:

(9) $$\begin{array}{c} {\text{Score}}_{\text{cosine}}\;\left(O_{\text{q}},O_{\text{a}}\right)=\frac{O_{\text{q}}\cdot O_{\text{a}}}{\left|O_{\text{q}}\right|\left|O_{\text{a}}\right|}, \end{array}$$

(10) $$\begin{array}{c} {\text{Score}}_{\text{Euclidean}}\;\left(O_{\text{q}},O_{\text{a}}\right)=\frac{1}{1+{\left\|O_{\text{q}}-O_{\text{a}}\right\|}_{2}}. \end{array}$$

Normalize the cosine similarity to the [0, 1] interval and it can be obtained as follows:

(11) $$\begin{array}{c} {\text{Score}}_{\text{cosine}}\;\left(O_{\text{q}},O_{\text{a}}\right)=0.5{\text{Score}}_{\text{cosine}}\;\left(O_{\text{q}},O_{\text{a}}\right)+0.5,\\ \text{Score }\left(O_{\text{q}},O_{\text{a}}\right)=\frac{2\cdot {\text{Score}}_{\text{cosine}}\;\left(O_{\text{q}},O_{\text{a}}\right)\cdot {\text{Score}}_{\text{Euclidean}}\left(O_{\text{q}},O_{\text{a}}\right)}{{\text{Score}}_{\text{cosine}}\;\left(O_{\text{q}},O_{\text{a}}\right)+{\text{Score}}_{\text{Euclidean}}\left(O_{\text{q}},O_{\text{a}}\right)}, \end{array}$$

where · represents the point multiplication operation, |*O*_q_| and |*O*_a_| represent the modulus length of the corresponding vector, respectively. ‖*O*_q_ − *O*_a_‖_2_ is the Euclidean distance between two points, and the values of equations (9) and (10) are in the range of [0, 1].

During training, the positive and the negative samples can be input simultaneously by using the hinge loss function. We define the hinge loss function as the training goal as below:

(12) $$\begin{array}{c} L=\max\;\left\{0,M-\text{Score}\left(O_{\text{q}},O_{\text{a}+}\right)+\text{Score}\left(O_{\text{q}},O_{\text{a}-}\right)\right\}+\lambda \left\|\theta \right\|, \end{array}$$

where *M* is the constant margin, a+ and a− denote the positive answer and the negative answer, respectively. *λ* and *θ* represent regularization parameters and neural networks parameters separately.

In the process of training, we utilize the backpropagation algorithm to calculate the gradient ∂*L*/∂*θ* and update the parameter *θ* to achieve the minimization of the objective function [34]. Finally, we update the parameters with the minimum objective function *L*_min_.

## 4. Experiments

In this section, we will introduce the detailed information of the experimental implementation, including TREC-QA (8-13) dataset and Wiki-QA dataset, model evaluation indicators, and selection of training parameters, and then, we will carefully analyze the experimental results on different datasets to prove that our proposed model has good accuracy and robustness.

### 4.1. Implementation Details

#### 4.1.1. Datasets

In this part, we mainly introduce two public datasets, TREC-QA (8-13) dataset and Wiki-QA dataset, and we also introduce the source, data characteristics, and the number of Q&A pairs of these two datasets in detail.

The experiment is operated on the Text REtrieval Conference 8-13 QA datasets (http://nlp.stanford.edu/mengqiu/data/qg-emnlp07-data.tgz) to evaluate our model, which was created by Wang et al. [35] and further elaborated by Yao et al. [17]. As shown in Table 1, we use the 53417 Q&A pairs in TREC 8-12 to train the model, while using 1148 Q&A pairs and 1517 Q&A pairs in TREC 13 for development and testing, respectively. Among them, per question in the development set contains 2.7 positive answers and 11.3 negative answers; per question in the test set contains 3.2 positive answers and 14.0 negative answers. Following Yao et al. [17], candidate answer sentences with more than 40 words and questions with only positive or negative candidate answer sentences are removed from the assessment.

Wiki-QA (https://www.microsoft.com/en-us/download/details.aspx?id=52419) is an open domain Q&A dataset provided by the Microsoft team in 2015. The questions in Wiki-QA are mainly focused on the question of classification, number, and personal information. They are collected and organized by real data of users. The candidate answer statement comes from the topmost text paragraph returned by the Wikipedia input page. As shown in Table 2, after filtering out the question without the correct answer, a total of 1242 Wiki-QA questions were obtained, and 293 correct answer sentences matched the problem, and the data format of Wiki corpus is not much different from TREC-QA (8-13).

In this paper, all experiments were performed on Python, MATLAB, and their optimization toolboxes on a computer with an Intel Core 2 Duo 2.93 GHz processor and a Windows 7 operating system.

#### 4.1.2. Evaluation Metrics

Following the previous works of Wang et al. [35] on this task, two evaluation metrics are utilized for our task: mean average precision (MAP) and mean reciprocal rank (MRR). MAP is the mean average precision score for each query. It reflects the performance of the retrieval system on all queries. The higher the order of related documents returned by the system, the larger the value of the corresponding MAP. MRR indicates the location of the first correct answer associated with the query. The more forward the answer stands, the larger the corresponding MRR value is. Higher values for MAP and MRR indicate better system performance. We resort the official trec_eval (http://trec.nist.gov/trec_eval/) scripts to calculate these metrics:

(13) $$\begin{array}{c} \text{MAP}=\frac{1}{N_{\text{q}}}\overset{N_{\text{q}}}{\underset{i=1}{\sum }}P_{i}\left(r\right),\\ P_{i}\left(r\right)=P_{i}\left(1\right)=\frac{1}{n_{ai}}\overset{n_{a_{i}}}{\underset{k=1}{\sum }}\frac{k}{{\text{rank}}_{k}},\\ \text{MRR}=\frac{\sum_{i=1}^{N_{\text{q}}}\left(1/{\text{rank}}_{i}\right)}{N_{\text{q}}}, \end{array}$$

where *N*_q_ represents the number of all queries and *n*_*ai*_ represents the number of all relevant correct answers for query *i*. *P*_*i*_(*r*) represents the average accuracy of the *i*-th query with recall ratio *r*. rank_*k*_ represents the position of the *k*-th correct candidate answer in the entire answer sequence after confidence ranking of the candidate answers for the query. rank_*i*_ represents the position in which the first correct candidate answer for query *i* is located in the set of candidate answers.

#### 4.1.3. Experimental Setting

In this paper, different experimental factors are set to test and evaluate our proposed method, and then our method is compared with other most advanced methods under the same dataset. The neural network model is implemented with TensorFlow library. In the course of training, we continuously observe the performance on the test set and select the highest MAP and MRR score parameters for final evaluation. Our implementation is as follows:

*(1) Word Embedding*. Pretrained GloVe (https://github.com/stanfordnlp/GloVe) [36] is used as the word embedding layer offered by the shared task with 400 dimensions. In addition, each sentence is padded with OOV (out of vocabulary) handling method to the maximum length of fixed lengths, which is 40 words for question and answer. In the candidate answer pool, we set the number of negative answers *K* = 5.

*(2) Parameter Initialization*. During training, we set the minimum batch size to 40 and refer to the Adam [13] experiment on the TensorFlow to initialize the learning rate to 0.001. The margin *M* is fixed to 0.2 and the regularization parameter *λ* is set to 1*e* − 5. Furthermore, we experimented with single-layer BiLSTM, stacked BiLSTM, and stacked BiLSTM with coattention. Each layer of LSTM has a memory size of 200.

*(3) Optimization Algorithm*. Adam algorithm [37] is resorted with the decay rate of 0.95 to update the parameters and optimize our model. Subsequently, we add dropout layer after word embedding to avoid overfitting and set dropout rate to 0.5. In order to effectively control the weights within a certain range to avoid gradient explosions, the clip gradients method is used and the gradient threshold is set to 5.

### 4.2. Results and Analysis

In order to verify the validity and accuracy of the algorithm model of the fusion stacked BiLSTM network and the coattention mechanism in the intelligent question answering, we tested and verified the TREC-QA (8-13) dataset and Wiki-QA dataset, respectively, and the experimental results were analyzed and summarized.

#### 4.2.1. Results and Analysis of TREC-QA (8-13) Dataset

**Figure 6:**
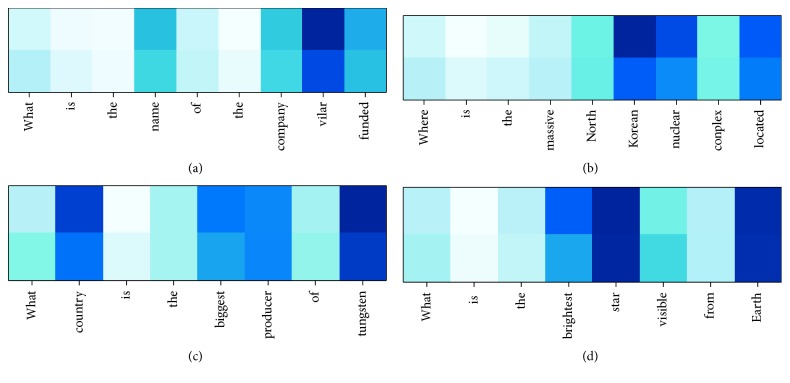
Comparison of sentence semantic analysis with or without coattention.

**Figure 7:**
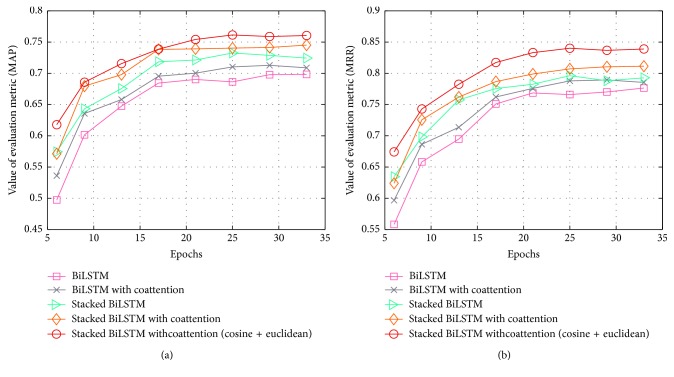
Variation in evaluation metrics with the epochs: (a) MAP and (b) MRR.

We conducted a comparative experiment on single-layer BiLSTM, stacked BiLSTM, and stacked BiLSTM with coattention on the TREC-QA (8-13) dataset.  Figure 6 compares the sentences of semantic analysis with or without coattention.  Figure 7 reveals the variation in evaluation metrics with the epochs.  Table 3 shows the details of experimental results for all mentioned baselines and our proposed model.

1. Different from the traditional work of Yih et al. [18] and Yu et al. [38], who analyzed the problem from the perspective of sentence structure, it can be obviously discovered that both our experiments and many previous studies such as BiLSTM [1] and CNN [39] have achieved better performance. These researches show that the semantic analyses of sentences are very necessary for NLP tasks and the deep neural networks are able to make the sentence vectors more representatives.
2. We found that our experimental results of the coattention mechanism were significantly better than most of the above results [1, 8, 38]. Specifically, comparing the results of line 15 with Nie et al. [8], our model achieved 3.52% gain for MAP and 3.83% gain for MRR. These experimental results strongly demonstrated that coattention mechanism and attention mechanism play an important role in improving NLP experimental results. The proper use of them allows the model to pay attention to the output vectors and extract the critical information well in the case of flexible input format. In this way, they can fix the lexical gap between questions and answers while capturing QA pair correlations.
3. The experimental index of stacked BiLSTM is better than single-layer BiLSTM when compared line 11 and line 12 with line 13 and line 14, respectively. Furthermore, Wang and Nyberg [6] resorted three-layer BiLSTM networks and achieved an increase in MAP (1.52%) and MRR (1.49%) over single-layer BiLSTM of line 11. In general, the appropriate amount of multilayer BiLSTM networks helps to understand the relationship between words and words in a deep level and better extract the characteristics of the sentence itself.
4. The best MAP (0.7613) and MRR (0.8401) are obtained by incorporating the coattention mechanism into a stacked BiLSTM neural networks and combining cosine similarity and Euclidean distance to calculate the matching degree between two vectors. Our experimental result outperforms the state-of-the-art baselines of Tan et al. [1, 7] by MAP (0.83%) and MRR (0.79%), respectively, which shows that combining the cosine similarity and the Euclidean distance balances the relationship between the angles and distances of two vectors to more effectively match questions and answers.

Firstly, we conducted comparative experiments in the model training process, selected the question and answer statement from the test set of TREC-QA (8-13) randomly, trained the model with/without coattention mechanism, and obtained the corresponding semantic vector representation through different models. The specific content verified that the presence or absence of a coattention mechanism had an impact on the analytical representation of the semantics of the statement. The comparison results are shown in Figure 6.

In Figure 6, the top row of the four matrices represents the semantic parsing results after the action of the coattention mechanism. The following line does not have this mechanism. It can be seen from the figure that after adding the coattention mechanism, the more critical words of the four sentences get more weights; they are more prominent in the process of parsing the expression of the statement, and the verbs such as “is” and “the.” The semantic weight ratio of the articles is correspondingly reduced. The analysis shows that the coattention mechanism has the ability to capture the relationship between the statement itself and the statement and can make the semantic expression of the statement more fully without adding additional artificial conditions.

Secondly, we verified the epoch sensitivity of the above several models under different iteration periods.  Figure 7 shows the variation in MAP and MRR for each model. We performed a comparative experiment of five models, including BiLSTM, stacked BiLSTM, stacked BiLSTM with coattention, BiLSTM with coattention, and stacked BiLSTM with coattention; furthermore, we also presented changes in MAP and MRR for the same model at different epochs.

We performed an epoch-number sensitivity analysis on our proposed model, which varied from 5 to 35.  Figure 7 displays the changes in the validation data for MAP and MRR when we change the number of epochs. We observed that both MAP and MRR changed with increasing the number of epochs but tended to be stable after epoch 25. However, the MAP and MRR values of some models have a decreasing trend as the epoch number increases more than 30. It reflects that a certain range of iterations is able to enhance the learning ability of the model and improve the experimental results.

We presented an optimized deep model by using stacked BiLSTM, coattention mechanism, attention mechanism, and a combined similarity metric, and our experimental results are shown in line 11 to line 15 of Table 3. We compared and summarized our observations as follows.

#### 4.2.2. Results and Analysis of Wiki-QA Dataset

We did further comparison experiments on the Wiki-QA dataset. Validation of the model on the Wiki-QA dataset makes the proposed approach more convincing. The parameter initialization and preset aspects of the model on the Wiki-QA dataset are basically consistent with the settings of the TREC dataset, where the batch size of the dataset is 30. Because it is also the order of information retrieval and candidate answer rankings, according to the official evaluation data, the evaluation metrics are selected as MAP and MRR.

**Figure 8:**
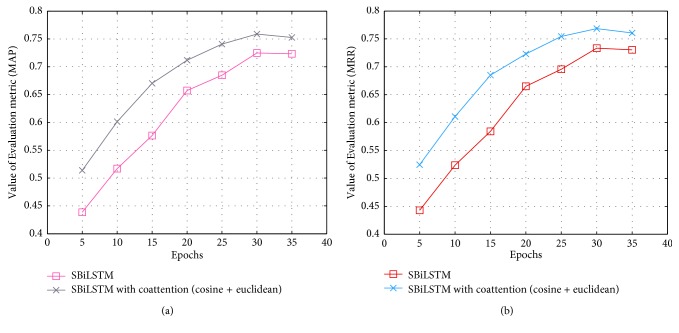
Variation in evaluation metrics with the epochs: (a) MAP and (b) MRR.

We also validated the various models of the design under different epochs on the Wiki-QA dataset, as shown in Figure 8. It can be seen from the figure that the model tends to be stable as the epoch reaches 30 times. When the number of epoch continues to increase, both MAP and MRR have a slight downward trend. The experimental results not only prove that the problem-solving of the model architecture analysis in this paper is effective for the sentence semantics, but also prove that the model has good accuracy and robustness.

The experimental results of each model under the Wiki-QA dataset are shown in Table 4. Compared with the current related research, the model results are superior to most baseline models [40, 41]. Comparing the results of line 1 and line 5 of Table 4, it can be seen that the stacked BiLSTM model is much more accurate than the single-layer LSTM model. In addition, the best experimental results of the model compared with the model in [42], the average accuracy is 0.05% higher than the model in [42].

In the field of intelligent question answering, these data results confirm that the model has some excellent performance in the statement semantic capture representation of questions and answers and can better represent semantic features.

## 5. Conclusion

In this paper, we proposed a stacked BiLSTM neural network based on the coattention mechanism for question answering. Stacked BiLSTM is used to sentence semantic understanding and modeling; coattention mechanism and attention mechanism are utilized to obtain the codependent representation of questions and answers; the combination of cosine similarity and Euclidean distance is used to calculate the similarity between the question and the answer. As reported in Section 4.2, we conduct experiments on the datasets of TREC-QA (8-13) and Wiki-QA, and then experiments on the TREC-QA (8-13) dataset demonstrated that the best MAP (0.7613) and MRR (0.8401) are achieved by using our model. We obtained a certain degree of improvement in MAP (0.83%) and MRR (0.79%) compared with other optimal baselines. Experimental results show that the proposed model is efficient for question answering. Note that, the experiment was only tested on two small datasets. The future work would focus on the implementation of replacing the original coattention mechanism with dynamic coattention network plus (DCN+) and incorporating CNN into the model to improve the experimental results. In addition, the implementation of the proposed model in other large-scale datasets such as SQuAD and SemEval-cQA will be an important issue for the next work.

## Figures and Tables

**Table 1:** Details of TREC-QA (8-13) dataset.

Set	Source	Questions	Positive answers	Negative answers	Length
Train-All	TREC 8-12	1229	6403	47014	≤40
Dev	TREC 13	84	222	926	≤40
Test	TREC 13	100	284	1233	≤40
Total	TREC 8-13	1411	6909	49173	≤40

**Table 2:** Details of Wiki-QA dataset.

Set	Questions	Positive answers	Negative answers	Length
Train-All	873	1040	19320	16.27
Dev	126	140	2593	15.91
Test	243	100	5872	16.11
Total	1242	293	27785	16.17

**Table 3:** Experimental results of different baselines and our proposed model on Train-All data.

Idx	Model	MAP	MRR
1	Probabilistic quasi-synchronous grammar [35]	0.6029	0.6852
2	Tree edit models [2]	0.6091	0.6917
3	Linear-chain CRF [17]	0.6307	0.7477
4	LCLR [18]	0.7092	0.7700
5	Bigram + count [38]	0.7113	0.7846
6	Three-layer BiLSTM + BM25 [6]	0.7134	0.7913
7	Convolutional deep neural networks [39]	0.7459	0.8078
8	BiLSTM/CNN with attention [7]	0.7111	0.8322
9	Attentive LSTM [1]	0.7530	0.8300
10	BiLSTM encoder-decoder with step attention [8]	0.7261	0.8018
11	BiLSTM	0.6982	0.7764
12	Stacked BiLSTM	0.7127	0.7893
13	BiLSTM with coattention	0.7325	0.7962
14	Stacked BiLSTM with coattention	0.7451	0.8114
15	Stacked BiLSTM with coattention (cosine + Euclidean)	**0.7613**	**0.8401**

**Table 4:** Experimental results of different baselines and our model on the Wiki-QA dataset.

Idx	Model	MAP	MRR
1	LSTM with attention [40]	0.6639	0.6828
2	CNN-Cnt [41]	0.6520	0.6086
3	wGRU-sGRU-Gl2 [42]	0.7537	0.7658
4	wGRU-sGRU-Gl2-Cnt [42]	**0.7638**	**0.7825**
5	Stacked BiLSTM	0.7248	0.7333
6	SBiLSTM-coA (cosine + Euclidean)	**0.7643**	**0.7751**

## Data Availability

This work involved data from the Text REtrieval Conference (TREC) 8-13 datasets and Wiki-QA datasets. We used the 53417 Q&A pairs in TREC 8-12 to train the model, while using 1148 Q&A pairs and 1517 Q&A pairs in TREC 13 for development and testing, respectively. All researchers can access the data in the following site: http://nlp.stanford.edu/mengqiu/data/qa-emnlp07-data.tgz, https://www.microsoft.com/en-us/download/details.aspx?id=52419. The data are divided into train data and development/test data.
